# A genetically-encoded chloride and pH sensor for dissociating ion dynamics in the nervous system

**DOI:** 10.3389/fncel.2013.00202

**Published:** 2013-11-13

**Authors:** Joseph V. Raimondo, Bradley Joyce, Louise Kay, Theresa Schlagheck, Sarah E. Newey, Shankar Srinivas, Colin J. Akerman

**Affiliations:** ^1^Department of Pharmacology, University of OxfordOxford, UK; ^2^UCT/MRC Receptor Biology Unit, Division of Medical Biochemistry, Faculty of Health Sciences, Institute of Infectious Disease and Molecular Medicine, University of Cape TownCape Town, South Africa; ^3^Department of Physiology, Anatomy and Genetics, University of OxfordOxford, UK

**Keywords:** genetic reporters, chloride sensors, pH sensors, intracellular chloride, intracellular pH, neural activity, fluorescence microscopy

## Abstract

Within the nervous system, intracellular Cl^−^ and pH regulate fundamental processes including cell proliferation, metabolism, synaptic transmission, and network excitability. Cl^−^ and pH are often co-regulated, and network activity results in the movement of both Cl^−^ and H^+^. Tools to accurately measure these ions are crucial for understanding their role under physiological and pathological conditions. Although genetically-encoded Cl^−^ and pH sensors have been described previously, these either lack ion specificity or are unsuitable for neuronal use. Here we present ClopHensorN—a new genetically-encoded ratiometric Cl^−^ and pH sensor that is optimized for the nervous system. We demonstrate the ability of ClopHensorN to dissociate and simultaneously quantify Cl^−^ and H^+^ concentrations under a variety of conditions. In addition, we establish the sensor's utility by characterizing activity-dependent ion dynamics in hippocampal neurons.

## Introduction

Chloride (Cl^−^) and hydrogen (H^+^) ions are fundamental to a wide range of processes within the nervous system including cell division, volume regulation, migration, metabolism, synaptic vesicle loading, network excitability, and fast synaptic inhibition (Tabb et al., 1992; Denker and Barber, 2002; Putney and Barber, 2003; Farrant and Kaila, 2007). Cl^−^ and H^+^ are often co-regulated (Russell and Boron, 1976; Kaila et al., 1989) and activity-dependent neuronal processes typically involve either the related, or independent, flux of both Cl^−^ and H^+^ ions (Chesler, 2003; Farrant and Kaila, 2007). Whilst intracellular Cl^−^ concentration ([Cl^−^]_i_) and the negative logarithm of intracellular H^+^ ion concentration (pH_i_) are known to affect network excitability, network activity itself can generate shifts in the intracellular concentrations of these two ions (Isomura et al., 2003; Raimondo et al., 2012b). This reciprocal relationship means that tools to accurately and independently measure [Cl^−^]_i_ and pH_i_ are important for understanding the separate and combined roles that these ions play during physiological and pathological network states.

Cl^−^ or pH sensitive microelectrodes generated some of the earliest measurements of [Cl^−^]_i_ and pH_i_. However, due to their size and potential effect upon cell integrity, the use of these microelectrodes has been most successful in large invertebrate neurons. Fluorescent dyes have also been widely used to report both Cl^−^ and pH. However, the most sensitive Cl^−^ dyes, such as N-(ethoxycarbonylmethyl)-6-methoxyquinolinium bromide (MQAE), are not ratiometric and so do not measure absolute Cl^−^ concentrations. In addition, these dyes tend to suffer from problems associated with toxicity, rapid bleaching, and cell leakage (Bregestovski et al., 2009). In contrast, the most popular pH sensitive dyes, such as 2′-7′-bis(carboxyethyl)-5(6)-carboxyfluoroscein (BCECF) and the seminaphthorhodafluors (SNARFs), are well tolerated and offer ratiometric estimation of absolute pH. Nonetheless, these dyes can interfere with endogenous H^+^ ion transport mechanisms and cannot be genetically targeted to cell types or subcellular compartments (Gatto and Milanick, 1993).

The discovery that green fluorescent protein (GFP) demonstrates inherent Cl^−^ and pH fluorescence sensitivity marked the beginning of an effort to develop genetically-encoded reporters of Cl^−^ or pH (Jayaraman et al., 2000; Kuner and Augustine, 2000). One approach has been to create fusions of two GFP mutants: yellow fluorescent protein (YFP) and cyan fluorescent protein (CFP). Whereas YFP emission is reduced by Cl^−^ or H^+^ binding, CFP fluorescence is relatively unaffected and serves as a fluorescence resonance energy transfer (FRET) donor for YFP. These fusion proteins are therefore useful as ratiometric reporters of either [Cl^−^]_i_ or pH_i_, and proteins within this group include Clomeleon, Cl-sensor, YFpH, and the pHlameleons (Kuner and Augustine, 2000; Awaji and Hirasawa, 2001; Esposito et al., 2008; Markova et al., 2008). Unfortunately, YFP's dual sensitivity to Cl^−^ and pH complicates the interpretation of measurements using these reporters and means that if the concentration of both ions change, it is not possible to dissociate the underlying ion fluxes. This is compounded by the fact that cellular processes often involve concomitant changes in both Cl^−^ and H^+^ ion concentration (Russell and Boron, 1976; Kaila et al., 1989). Whilst other genetically-encoded pH indicators have been developed, such as pHlourin (Miesenböck et al., 1998) (RaGFP) and the deGFPs (Hanson et al., 2002), their susceptibility to artifacts based on Cl^−^ sensitivity has not been well-characterized.

This issue was addressed by the introduction of ClopHensor (Arosio et al., 2010)—a fusion protein that is able to simultaneously measure concentrations of both Cl^−^ and H^+^ ions and has been shown to function in heterologous cell lines (Arosio et al., 2010; Mukhtarov et al., 2013). ClopHensor is composed of the Cl^−^ and pH sensitive GFP mutant, E^2^GFP, fused to monomeric DsRed. Here we identify problems associated with the use of ClopHensor in the nervous system. We therefore re-engineer and improve this reporter, and present “ClopHensorN,” a new, genetically-encoded ratiometric Cl^−^ and pH sensor that is optimized to dissociate ion dynamics in neuronal cell types. We demonstrate the ability of ClopHensorN to simultaneously quantify Cl^−^ and pH fluxes under a variety of conditions. Expression of ClopHensorN in hippocampal neurons enables us to dissect changes in intracellular Cl^−^ and pH in response to network activity, which would not be possible with previous reporters.

## Materials and methods

### DNA constructs and subcloning

The original ClopHensor construct (Arosio et al., 2010), and ClopHensor fused to two palmitoylation sites (“PalmPalm-ClopHensor”), were kindly provided by Daniele Arosio (University of Trento; Addgene plasmids 25938 and 25940). ClopHensor was moved into the expression vector pBJ1 under the control of a chicken beta actin (CAG) promoter and the DsRed monomer was replaced with either mCherry or tandem dimer tomato (tdTomato). The new constructs maintained the 20 amino acid linker sequence RGSASGGGGGLVPRGSASGA between E^2^GFP and the red fusion protein, with the addition of two amino acids (TG) at the C-terminal end of the linker. Western blotting experiments confirmed that both the original ClopHensor and the new ClopHensorN construct generated >90% full-length fusion proteins (data not shown). Upon publication the constructs presented here will be made freely available from the non-profit service Addgene (http://www.addgene.org/).

### Slice preparation and DNA transfection

Rat organotypic hippocampal slice cultures were prepared using a method similar to that described by Stoppini et al. (1991). Briefly, 7 day old male Wistar rats were killed in accordance with the UK Animals Scientific Procedures Act 1986. The brains were extracted and placed in cold (4°C) Geys Balanced Salt Solution (GBSS), supplemented with D-glucose (34.7 mM). All reagents were purchased from Sigma-Aldrich, unless stated. The hemispheres were separated and individual hippocampi were removed and immediately sectioned into 350 μm thick slices on a McIlwain tissue chopper. Slices were rinsed in cold dissection media, placed onto Millicell-CM membranes and maintained in culture media containing 25% EBSS, 50% MEM, 25% heat-inactivated horse serum, glucose, and B27 (Invitrogen). Slices were incubated at 36°C in a 5% CO_2_ humidified incubator before transfection. Neurons were biolistically transfected after 4–7 days *in vitro* using a Helios Gene Gun (120 psi; Bio-Rad). Fifty microgram of target DNA was precipitated onto 25 mg of 1.6 μm diameter gold microcarriers and bullets generated in accordance with the manufacturer's instructions (Bio-Rad). Biolistic delivery of target DNA resulted in sparse transfection rates (typically less than 10 cells per slice) and recordings were performed 2–7 days post-transfection. For a subset of experiments ClophensorN was expressed in mouse cortex by *in utero* electroporation. *In utero* electroporation was carried out on E14.5 C57BL6/J (Jackson Laboratory) mouse embryos. Dams were anesthetized using isoflorane (3% for induction, 2–2.5% for surgery) and the uterine horns exposed by laparotomy. Each embryo was injected through the uterine wall with 0.5–1 ul ClopHensorN plasmid (2 ug/ul) in PBS with 0.03% fast green (Sigma) using a thin-walled glass pipette (WPI) pulled to a ~50 μm tip. Paddle electrodes (Nepagene, CUY650) were used to deliver five 50 ms, 42 V pulses at 1 Hz from a square pulse generator (BTX, ECM 830). Embryos were kept moist during the surgery by applying warm sterile PBS. Following electroporation the uterine horns were replaced and the dam allowed to recover and litter as normal. At postnatal day 21 the mice were killed and the brain rapidly removed and placed in ice-cold (0 to +4°C) artificial cerebro-spinal fluid (ACSF), bubbled with 95% O_2_/5% CO_2_. Coronal cortical slices (350–400 μm thickness) were cut using a vibrating microtome (Microm HM650V, Carl Zeiss Ltd) and slices were maintained in an interface chamber between humidified carbogen gas (95% O_2_, 5% CO_2_) and ACSF (at 20–25°C). After recovering for at least 1 h, the slices were mounted on coverslips (coated with 0.1% poly-L-lysine in ultrapure H_2_O) before being transferred to the recording chamber for imaging.

### Electrophysiological recordings and activity-dependent manipulations

Organotypic hippocampal slices or acute cortical slices were transferred to a recording chamber and continuously superfused with 95% O_2_/5% CO_2_ oxygenated ACSF, warmed to 32–35°C. The composition of the “standard” ACSF was (in mM): NaCl (120), KCl (3), MgCl_2_ (2), CaCl_2_ (2), NaH_2_PO_4_ (1.2), NaHCO_3_ (23), D-Glucose (11). The pH was adjusted to be between 7.35 and 7.40 using NaOH. Synchronous network activity was induced by switching bath perfusion of slices with normal ACSF to nominally Mg^2+^-free ACSF (Anderson et al., 1986) (Mg^2+^ omitted from standard ACSF) or nominally Cl^−^-free ACSF (Yamamoto and Kawai, 1967) (NaCl, MgCl_2_ and CaCl_2_ of standard ACSF replaced with 120 mM sodium D-gluconate, 1 mM MgSO_4_ and 3 mM calcium D-gluconate, respectively). Patch pipettes of 3–5 MOhm tip resistance were pulled from filamental borosilicate glass capillaries (1.2 mm outer diameter, 0.69 mm inner diameter; Harvard Apparatus Ltd), using a horizontal puller (Sutter P-97). For whole-cell recordings, pipettes were filled with an internal solution containing (in mM): K-gluconate (130), NaCl (10), CaCl_2_ (0.1333), MgCl_2_ (2), EGTA (1), KCl (4), and HEPES (10). For the GABA_A_ receptor activation experiments a cesium-based internal solution was used containing (in mM): cesium gluconate (120), 40 mM HEPES (40), NaCl (4), ATP-Mg (2), Na-GTP (0.3), MQX-314 (0.2) and biocytin (4 mg/ml). The osmolarity of internal solutions was adjusted to 290 mOsM and the pH was adjusted to 7.38 with KOH.

Pyramidal neurons within the CA1 and CA3 regions were visualized under a 40× water-immersion objective (Leica) and targeted for recording. Patch-clamp recordings were made using an Axopatch 1D or Axoclamp 2B amplifier (Axon Instruments). Data was acquired with WinWCP Strathclyde Whole Cell Analysis software (V.3.9.7; University of Strathclyde) before being exported to the MATLAB environment (MathWorks) for further analysis using customized scripts. Some statistical analysis was performed using GraphPad Prism version 5.00 (GraphPad Software). Data are reported as mean ± SEM.

GABA_A_ receptors were activated either by exogenous application of GABA or by electrical stimulation of GABAergic afferents. Short “puffs” of GABA (200 μM) were applied via patch pipette positioned close to the soma and connected to a picospritzer (20 psi for 20 ms; General Valve). Synaptic GABA_A_ receptor activation was achieved by stimulating afferents using a bipolar stimulating electrode (Frederick Haer Company) placed in stratum radiatum, 300–400 μm from the recorded cell (Pouille and Scanziani, 2001). Simultaneous activation of glutamatergic receptors was prevented using bath application of kynurenic acid (2 mM).

### Imaging and calibrating intracellular Cl^−^ and pH

**Figure 1:**
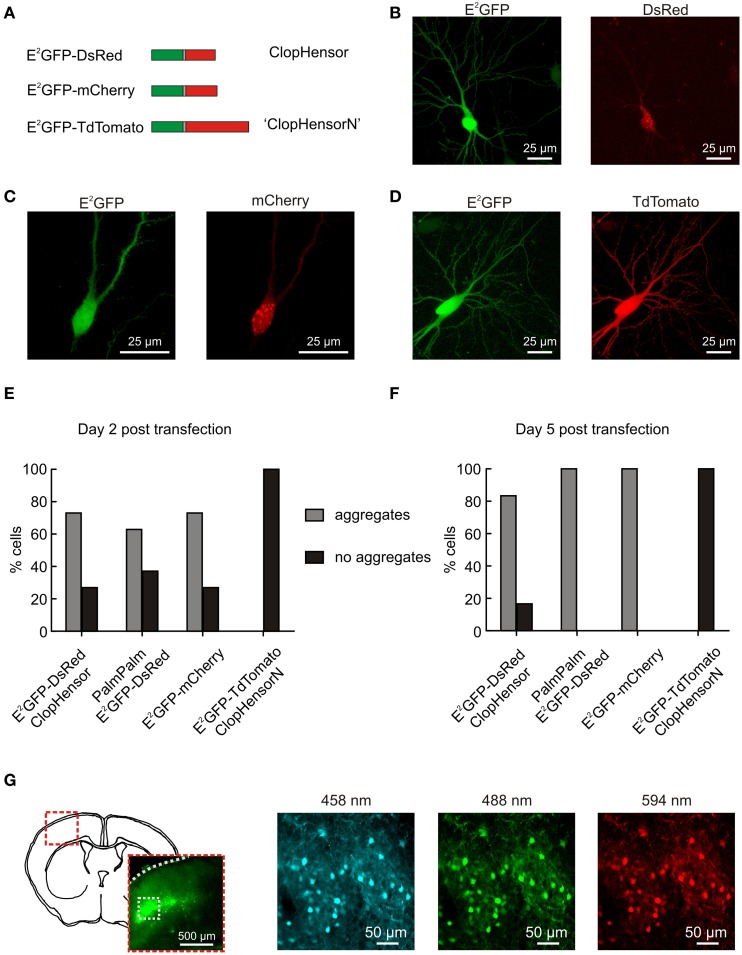
**ClopHensorN: a genetically-encoded Cl^−^ and pH sensor optimized for use in the nervous system. (A)** Biolistic transfection of hippocampal slices was used to test different fusion proteins for use as a simultaneous Cl^−^ and pH sensor in the nervous system. These were composed of E^2^GFP (green) fused to one of three different red fluorescent proteins (RFPs; red) via a flexible linker (gray). The RFP was either DsRed (“ClopHensor”; top), mCherry (middle), or TdTomato (“ClopHensorN”; bottom). **(B)** A hippocampal CA3 pyramidal neuron expressing ClopHensor. The E^2^GFP (left) shows uniform expression but the DsRed (right) revealed marked intracellular aggregation and poor co-localization with the E^2^GFP. **(C)** A CA3 pyramidal neuron expressing the E^2^GFP-mCherry fusion protein. Pronounced aggregation issues also affect this construct. **(D)** A CA3 pyramidal neuron expressing the E^2^GFP-TdTomato fusion protein “ClopHensorN.” ClopHensorN displayed homogenous expression of both E^2^GFP and TdTomato, and complete co-localization of the two fluorophores. **(E)** Population data from hippocampal slices 2 days after biolistic transfection with four different ClopHensor variants; E^2^GFP-DsRed (original ClopHensor), PalmPalm-E^2^GFP-DsRed (ClopHensor with a membrane targeting sequence—PalmPalm-ClopHensor), E^2^GFP-mCherry and E^2^GFP-TdTomato (ClopHensorN). The majority of cells expressing E^2^GFP-DsRed (19 of 26 cells imaged), PalmPalm E^2^GFP-DsRed (39 of 62 cells), and E^2^GFP-mCherry (19 of 26 cells) demonstrated dense aggregation of the red fluorophore. In contrast, no aggregates were detected in any of the cells expressing ClopHensorN (0 of 58 cells) and the two fluorophores exhibited uniform and corresponding expression. **(F)** 5 days post transfection, almost all cells expressing E^2^GFP-DsRed (20 of 24 cells), PalmPalm E^2^GFP-DsRed (24 of 24 cells) and E^2^GFP-mCherry (50 of 50 cells) contained dense aggregates. In contrast, all cells expressing ClopHensorN (0 of 36 cells with dense aggregates) continued to demonstrate uniform expression. **(G)** *In utero* electroporation of ClopHensorN in mouse embryos resulted in long-term and uniform expression in cortical neurons. After 4 weeks *in vivo*, expression was assessed using acute brain slices. A schematic of a coronal mouse brain slice at P21 and low magnification fluorescent image (left) indicate the position of the ClopHensorN-expressing cortical cells. High magnification confocal images collected following excitation at 458, 488, and 594 nm, respectively (right), show that ClopHensorN displays homogenous expression and co-localization of the two fluorophores in mature cortical neurons.

Imaging was performed using an upright Leica SP2 AOBS laser scanning confocal microscope equipped with a 40x water immersion objective (NA 0.8). To determine whether expression of ClopHensor and ClopHensor-derived constructs (including PalmPalm-ClopHensor) resulted in corresponding expression of E^2^GFP and the fused red fluorophore (either DsRed, mCherry or TdTomato), cells were excited at 488 nm (for E^2^GFP, “green channel”) and at 594 nm (for the red fluorophores, “red channel”). Emission was collected by separate photomultiplier tubes (PMTs): between 500 and 550 nm for the green channel and between 650 and 700 nm for the red channel. In a blinded manner, cells expressing each construct were counted. The number of cells demonstrating aggregates (examples Figures 1B,C) as a fraction of total expressing cells was recorded. Cells were counted as aggregated if there was an area of increased red fluorescence that was not matched by an increase in green fluorescence.

**Figure 2:**
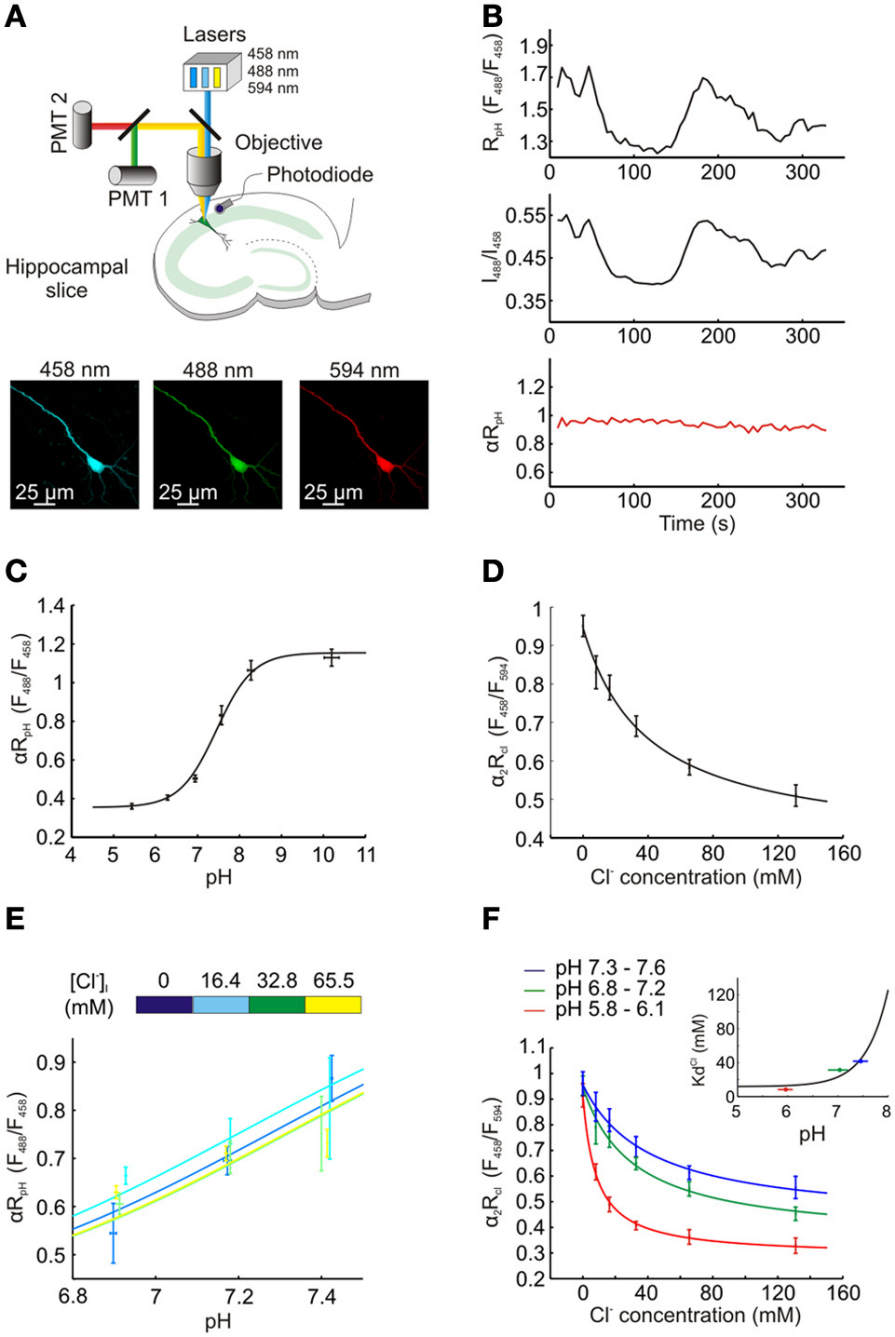
**Measuring intracellular Cl^−^ and pH concentration using ClopHensorN. (A)** Top, a schematic showing the experimental setup in which hippocampal pyramidal neurons expressing ClopHensorN were imaged. To determine [Cl^−^]_i_ and pH_i_, confocal images were collected following excitation at 458, 488, and 594 nm, respectively (bottom). **(B)** Lasers exhibit fluctuations over seconds and minutes, which affects real-time ratiometric imaging by excitation and requires offline correction, as described previously (Zucker and Price, 2001; Arosio et al., 2010). A hippocampal neuron expressing ClopHensorN was imaged in the presence of ionophores and an ACSF that “clamps” intracellular pH (pH_i_) and intracellular Cl^−^ concentration ([Cl^−^]_i_) at 7.8 pH units and 8 mM, respectively, (see Materials and Methods). Despite pH being held at this constant value, significant apparent changes in R_pH_ (F_488_/F_458_) were recorded over a period of 330 s (top trace). A photodiode placed below the tissue was used to continuously record the emission power of the lasers, which were then used to calculate the intensity ratio for the 488 laser and the 458 laser (I_488_/I_458_, middle trace). Note the strong correlation between the fluctuations in this ratio and R_pH_. Bottom trace (red), R_pH_ was corrected offline by a factor α, where α = 0.5 ÷ (I_488_/I_458_). This resulted in a stable estimate of pH under these “clamped” conditions and confirmed the efficacy of this correction process. In all experiments αR_pH_ was used to determine intracellular pH, and in a similar manner, α_2_ was used to correct R_Cl_ in order to determine [Cl^−^]_i_ (see Materials and Methods). **(C)** ClopHensorN calibration curve relating pH_i_ to the pH sensitive excitation fluorescence ratio (αR_pH_), following correction for laser power fluctuations. pH_i_ was manipulated by adjusting extracellular pH in the presence of a H^+^ permeable ionophore, data was fit using established equations (Grynkiewicz et al., 1985; Arosio et al., 2010) (see Equation 2, Materials and Methods) and pK_A_ was found to be 7.45 with a fitted 95% confidence interval (CI) between 7.29 and 7.62. **(D)** ClopHensorN calibration curve relating [Cl^−^]_i_ to the Cl^−^ sensitive excitation fluorescence ratio (α_2_R_Cl_), following correction for laser power fluctuations. [Cl^−^]_i_ was systematically varied by controlling extracellular Cl^−^ in the presence of a Cl^−^ permeable ionophore, data was fit using established equations (see Equation 5, Materials and Methods) and K_d_ found to be 39.2 mM with a fitted 95% CI between 11.7 and 66.7 mM. Error bars indicate SEM. **(E)** Varying [Cl^−^]_i_ within the physiological range had no effect on αR_pH_. **(F)** Consistent with previous studies using E^2^GFP (Bizzarri et al., 2006; Arosio et al., 2010), pH changes influence the affinity of ClopHensorN for Cl^−^. K^Cl^_d_ shifted from 8.3 mM with a fitted 95% CI between 3.6 and 13.0 mM (pH range 5.8–6.1) to 31.3 mM and 41.7 mM at pH ranges 6.8–7.2 and 7.3–7.6, respectively. The fitted 95% CI intervals for these K_d_s were 4.9 to 57.8 mM and −2.3 to 85.7 mM. The inset demonstrates the calculated dependence of K^Cl^_d_ on pH (using Equation 6, See Materials and Methods). The 3 K^Cl^_d_ values for the 3 pH ranges (horizontal bars) are shown. Utilizing this calibration data, ClopHensorN could be used to accurately and simultaneously report [Cl^−^]_i_ and pH_i_.

For intracellular Cl^−^ and pH imaging ClopHensorN was used as a ratiometric indicator by excitation and was excited sequentially at 458, 488, and 594 nm. Emission was collected between 500 and 550 nm with a single PMT when excited at 458 and 488 nm, but between 650 and 700 nm with a second PMT when excited at 594 nm. Images were exported to the MATLAB programming environment where background was subtracted and fluorescence averaged within regions of interest selected from the soma of individual cells. To correct for fluctuations in laser intensity, a photodiode (sample rate 10 kHz) recorded laser power output during imaging (Zucker and Price, 2001; Arosio et al., 2010) and the resulting data was used to correct fluorescence ratios offline by a factor α (for R_pH_) and α_2_ (for R_Cl_), see Figure 2B.

$$\begin{array}{l} \;\alpha =0.5÷\left(I_{488}/I_{458}\right)\\ \text{and }\alpha_{2}=0.5÷\left(I_{458}/I_{594}\right) \end{array}$$

where *I*_458_, *I*_488_, and *I*_594_ are the laser powers measured by the photodiode for excitation with the 458, 488, and 594 nm lasers, respectively.

In order to determine pH_i_ using ClopHensorN, laser power corrected excitation fluorescence ratios (αR_pH_) were measured as follows:

$$\alpha {\text{R}}_{\text{pH}}=F_{488}/F_{458}$$

*F*_488_ and *F*_458_ are the fluorescence recorded using excitation with the 488 and 458 nm lasers. The formation of a 1:1 analyte-sensor complex results in an equilibrium described by the Grynkiewicz equation (Grynkiewicz et al., 1985; Arosio et al., 2010), which can be written for ClopHensorN as follows:

(1) $${\text{pH}}_{\text{i}}={\text{pK}}_{\text{a}}+\log\left(\frac{\alpha {\text{R}}_{\text{pH}}-\alpha {\text{R}}_{\text{A}}}{\alpha {\text{R}}_{\text{B}}-\alpha {\text{R}}_{\text{pH}}}\right)+\log\left(\frac{F_{458,\text{A}}}{F_{458,\text{B}}}\right)$$

α*R*_*A*_ and α*R*_*B*_ are the values of αR_pH_ for ClopHensorN in its most acidic and basic forms, respectively. Likewise, *F*_458,A_ and *F*_458,B_ reflect the emission upon excitation at 458 nm, when the ratiometric indicator is in its acidic and basic form. pK_A_ is the acid dissociation constant of the indicator. Due to the fact that ClopHensorN has a pH isobestic point at 458 nm, fluorescence is pH insensitive when excited at this wavelength. That is, *F*_458,A_ = *F*_458,B_. Calibration data was therefore fitted using the following rearranged version of Equation 1:

(2) $$\alpha {\text{R}}_{\text{pH}}=\frac{\alpha {\text{R}}_{\text{B}}10^{\text{pH}-{\text{pK}}_{\text{A}}}+\alpha {\text{R}}_{\text{A}}}{1+10^{{\text{pH-pK}}_{\text{A}}}}$$

this allowed the pKa of ClopHensorN to be determined and pH_i_ to be calculated from measured fluorescence ratios (αR_pH_) during subsequent experiments.

Having determined pH_i_, [Cl^−^]_i_ could be calculated using the laser power corrected excitation fluorescence ratio (α_2_R_Cl_)

$$\alpha_{2}{\text{R}}_{\text{Cl}}=F_{458}/F_{594}$$

respectively. The Grynkiewicz equation for using ClopHensorN as a Cl^−^ indicator can be written as:

(3) $${\left[{\text{Cl}}^{-}\right]}_{\text{i}}={\text{K}}_{\text{d}}^{\text{Cl}}\left[{\text{pH}}_{\text{i}}\right]\text{​}\left(\frac{\alpha_{2}{\text{R}}_{\text{Cl}}-\alpha_{2}R_{\text{free}}}{\alpha_{2}R_{\text{bound}}\left[{\text{pH}}_{\text{i}}\right]-\alpha_{2}{\text{R}}_{\text{Cl}}}\right)\text{​}\left(\frac{F_{594,\text{free}}}{F_{594,\text{bound}}}\right)\;$$

*F*_594,free_ and *F*_594,bound_ reflect the fluorescence after excitation with the 594 nm when the ClopHensorN is in its Cl^−^ free and Cl^−^ bound forms. However, as the fluorescence of TdTomato is insensitive to Cl^−^, $\frac{F_{594,\text{free}}}{F_{594,\text{bound}}}=1$ and Equation 3 can be simplified to:

(4) $${\left[{\text{Cl}}^{-}\right]}_{\text{i}}={\text{K}}_{\text{d}}^{\text{Cl}}\left[{\text{pH}}_{\text{i}}\right]\left(\frac{\alpha_{2}{\text{R}}_{\text{Cl}}-\alpha_{2}R_{\text{free}}}{\alpha_{2}R_{\text{bound}}\left[{\text{pH}}_{\text{i}}\right]-\alpha_{2}{\text{R}}_{\text{Cl}}}\right)$$

*K*^Cl^_d_[pH_i_] is the Cl^−^ dissociation constant, which is dependent on pH (Arosio et al., 2010). α_2_*R*_free_ is the maximum value of α_2_*R*_Cl_, reflecting α_2_*R*_Cl_ where no Cl^−^ is bound to ClopHensorN. In contrast, α_2_*R*_bound_[pH_i_] is the minimum value of α_2_*R*_Cl_, reflecting α_2_*R*_Cl_ when ClopHensorN is saturated with Cl^−^. As demonstrated in Figure 2F, α_2_*R*_bound_[pH_i_] was also found to depend on pH. Calibration data was then fitted using the following rearranged version of Equation 4:

(5) $$\alpha_{2}{\text{R}}_{\text{Cl}}=\left(\frac{{\left[{\text{Cl}}^{-}\right]}_{\text{i}}\alpha_{2}R_{\text{bound}}\left[{\text{pH}}_{\text{i}}\right]+K_{\text{d}}^{\text{Cl}}\left[{\text{pH}}_{\text{i}}\right]\alpha_{2}R_{\text{free}}}{K_{\text{d}}^{\text{Cl}}\left[{\text{pH}}_{\text{i}}\right]+{\left[{\text{Cl}}^{-}\right]}_{\text{i}}}\right)$$

This allowed *K*^Cl^_d_[pH_i_], α_2_*R*_free_ and α_2_*R*_bound_[pH_i_] to be determined by performing Cl^−^ calibrations at different pH_i_ values (see Figure 2F). *K*^Cl^_d_[pH_i_] can be described according to the following equation (Arosio et al., 2007, 2010):

(6) KdCl[pHi]=K1dCl(1+10(pKA−pHi)10(pKA−pHi))

^1^K^Cl^_d_ reflects the Cl^−^ dissociation constant when ClopHensorN is in its most acidic form (fully protonated) and was determined by fitting Equation 5 with K^Cl^_d_[pH_i_] data derived from Equation 4. This allowed K^Cl^_d_[pH_i_] to be determined for any pH_i_. α_2_*R*_free_ was constant irrespective of pH_i_. The relationship between α_2_*R*_bound_[pH_i_] and pH_i_ was assumed to be linear and also fit using data from Equation 4.

(7) $$\alpha_{2}R_{\text{bound}}\left[{\text{pH}}_{\text{i}}\right]=M({\text{pH}}_{\text{i}})+\alpha_{2}R_{\text{bound}},{\text{pH}}_{0}$$

No weighting was used during the fitting procedures. Calibration data was acquired from ClopHensorN-expressing cells in hippocampal slices. Intracellular pH and Cl^−^ were controlled by equilibrating extra- and intracellular ion concentrations using the K^+^/H^+^ exchanger nigericin (10 μM) and the Cl^−^/OH^−^ exchanger tributyltinchloride (10 μM) in a high K^+^ containing ACSF, according to the method described previously by Boyarsky et al. (1988). ACSF of different [Cl^−^] were made by mixing two HEPES buffered ACSF solutions containing 0 mM or 131 mM Cl^−^, respectively. The 0 mM Cl^−^ solution contained (in mM): potassium D-gluconate (123), HEPES (23), D-glucose (11), NaH_2_PO_4_ (1.2), MgSO_4_ (2) and calcium D-gluconate (2). The 131 mM Cl^−^ solution contained (in mM): KCl (123), HEPES (23), D-glucose (11), NaH_2_PO_4_ (1.2), MgCl_2_ (2) and CaCl_2_ (2). Using these two solutions high K^+^ containing ACSF solutions of the following Cl^−^ concentrations were made: 131, 65.5, 32.75, 16.375, 8 mM, and 0 mM. pH was adjusted with small aliquots of NaOH and, to avoid CO_2_-dependent pH buffering, ACSF was bubbled with 100% O_2_. After each adjustment of either Cl^−^ or pH, at least 15 min were allowed for intracellular and extracellular compartments to equilibrate. For calibration purposes, this allowed the measurement of αR_pH_ and α_2_*R*_Cl_ at different, known [Cl^−^]_i_ and pH_i_. For all experimental data, (i.e., for each time point of a trace), pH_i_ was first determined from αR_pH_ using Equation 1. Using pH_i_, K^Cl^_d_[pH_i_] and *R*_bound_[pH_i_] were then calculated from Equations 6 and 7. Finally, this allowed [Cl^−^]_i_ to be determined using α_2_*R*_Cl_ and Equation 4 (see Results).

## Results

### ClopHensorN: a genetically-encoded Cl^−^ and pH sensor optimized for use within the nervous system

Recently Arosio et al. (2010) engineered a novel fusion protein capable of independently and simultaneously measuring Cl^−^ and pH. Named ClopHensor, this reporter is based on the fusion of a well-described pH and Cl^−^ sensitive GFP mutant E^2^GFP (Bizzarri et al., 2006), with the pH and Cl^−^ insensitive monomer DsRed (Figure 1A). The corresponding intracellular expression of E^2^GFP and DsRed is critical to ClopHensor's function as a ratiometric reporter of intracellular ion concentration. To test whether ClopHensor could be used to measure Cl^−^ and H^+^ in neurons, rat hippocampal brain slices were biolistically transfected with ClopHensor-expressing plasmids (Figure 1). Expression of ClopHensor or PalmPalm-ClopHensor in hippocampal pyramidal neurons often resulted in uniform E^2^GFP expression, but highly heterogeneous DsRed expression that occurred as dense intracellular aggregations (Figures 1B,E,F). This was associated with compromised cellular morphology and is consistent with previous reports that DsRed is susceptible to aggregation, particularly within fusion proteins (Shaner et al., 2005). In an attempt to address this issue we designed new fusion proteins where the DsRed monomer in the original ClopHensor was replaced with either mCherry or tandem dimer tomato (TdTomato; Figures 1A,C,D). Interestingly, like the original ClopHensor, the E^2^GFP-mCherry fusion exhibited intracellular aggregations and a lack of co-localization between the two fluorophores (Figures 1C,E,F). In contrast, the E^2^GFP-tdTomato fusion protein exhibited uniform and corresponding expression between the two fluorophores (Figures 1D–F). Whilst the other fusion proteins showed aggregations in more than 60% of the cells examined, this was not the case in any of the E^2^GFP-tdTomato-expressing cells (Figures 1E,F). To further confirm that E^2^GFP-tdTomato is well-tolerated, we delivered the construct *in vivo* by *in utero* electroporation and confirmed that this resulted in uniform and widespread expression in mature cortical neurons (Figure 1G). Together these data confirm that the E^2^GFP-tdTomato construct is well-suited for studies in the nervous system and has the potential to afford ratiometric simultaneous reporting of neuronal Cl^−^ and H^+^. We have named this new fusion protein “ClopHensorN.”

### ClopHensorN allows independent measurement of intracellular Cl^−^ and pH

We then assessed the ability of ClopHensorN to independently report steady-state Cl^−^ and pH in hippocampal pyramidal neurons. ClopHensorN was used as a ratiometric indicator of Cl^−^ and pH by excitation (Arosio et al., 2010) and was excited sequentially via excitation at 458, 488 and 594 nm using a confocal microscope (Figure 2A). To compensate for fluctuations in laser intensity, a photodiode was used to record laser power output during imaging (Zucker and Price, 2001; Arosio et al., 2010) and the resulting data was used to correct fluorescence ratios offline by a factor α (for R_pH_) and α_2_ (for R_Cl_) (see Figure 2B and Materials and Methods). Calibration of the reporter was performed by systematically varying extracellular Cl^−^ and pH in the presence of H^+^ and Cl^−^ permeable ionophores, which are known to equilibrate intra- and extracellular concentrations of these two ions. αR_pH_ was shown to depend upon intracellular pH with a pKa of 7.45 (Figure 2C). Importantly, systematically varying the intracellular Cl^−^ across the physiological range (0–65.5 mM), did not affect the αR_pH_ measurements. There was no correlation between intracellular Cl^−^ and αR_pH_ for the three pH values tested (for pH 6.85–6.94, *P* = 0.15, for pH 7.14–7.22, *P* = 0.73 and for pH 7.14–7.22, *P* = 0.11, Pearson Correlation, Figure 2E). Thus, consistent with previous work, E^2^GFP and hence ClopHensorN provides a Cl^−^ insensitive readout of pH (Bizzarri et al., 2006; Arosio et al., 2010; Raimondo et al., 2012b). In order to report Cl^−^, ClopHensor derived proteins make use of the fact that E^2^GFP possesses a pH isobestic point at 458 nm (Arosio et al., 2010). Fluorescence emission at this excitation wavelength is Cl^−^ sensitive, but not pH sensitive. Like DsRed, TdTomato's insensitivity to both Cl^−^ and pH (Shaner et al., 2004) make it a suitable partner for ratiometric imaging. ClopHensorN was therefore used to report Cl^−^ by calculating the ratio of fluorescence emission when E^2^GFP was excited at 458 nm, over the emission when TdTomato was excited at 594 nm (R_Cl_ = F_458_/F_594_) and corrected for laser power fluctuations to generate α_2_R_Cl_. Consistent with our predictions, α_2_R_Cl_ was strongly sensitive to [Cl^−^]_i_ (Figure 2D). In agreement with previous studies using E^2^GFP, pH changes influenced the affinity of ClopHensorN for Cl^−^(Bizzarri et al., 2006; Arosio et al., 2010), which could be accounted for by using the family of calibration curves (Figure 2F; see Materials and Methods). At low pH, (range 5.8–6.1), whereby the sensor approaches full protonation, the affinity for Cl^−^ is high with a K^Cl^_d_ of 8.3 mM. Due to the fact that E^2^GFP requires a proton to be bound in order to bind Cl^−^, at higher intracellular pH the affinity of E^2^GFP for Cl^−^ drops (Arosio et al., 2010). Indeed, at more physiological pH levels K^Cl^_d_ was 31.3 mM (pH range 6.8–7.2) and 41.7 mM (pH range 7.3–7.6). In summary, ClopHensorN allowed absolute neuronal Cl^−^ concentration and pH to be determined simultaneously from fluorescence ratios (αR_Cl_ and α_2_R_pH_), in a manner that is independent of protein expression levels. This was achieved by: (1) calculating pH in a region of interest from αR_pH_, (2) using this value to calculate the pH-dependent Cl^−^ binding parameters of ClopHensorN, and (3) using these parameters to calculate [Cl^−^]_i_ from α_2_R_Cl_. This method establishes ClopHensorN as an important new tool for quantifying Cl^−^ and H^+^ concentrations in neurons.

### Functional dissociation of intracellular Cl^−^ and pH measurements with ClopHensorN

**Figure 3:**
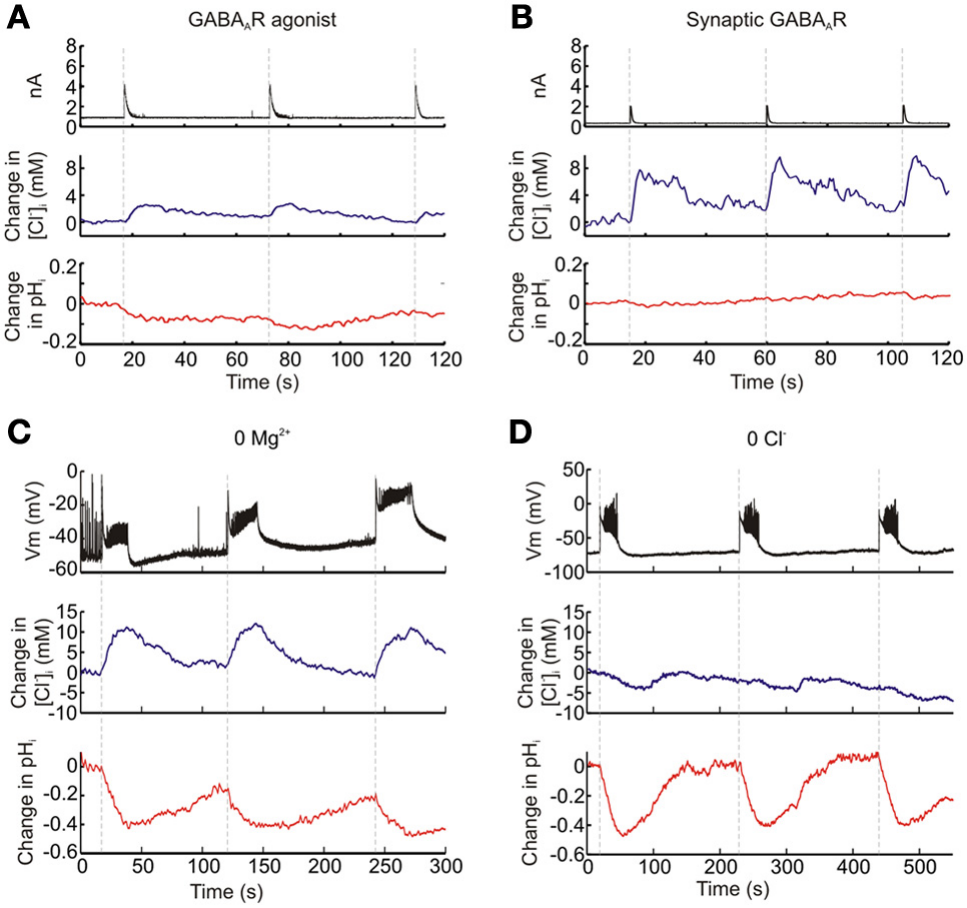
**Functional dissociation of intracellular Cl^−^ and pH with ClopHensorN**. Hippocampal pyramidal neurons expressing ClopHensorN were imaged whilst simultaneous patch clamp recordings were performed either from the ClopHensorN-expressing cell (“a” and “b”) or a neighboring pyramidal neuron (“c” and “d”). **(A)** Eliciting GABA_A_ receptor currents (black trace, top) via focal delivery of GABA (200 μM, 20 ms) resulted in both transient increases in [Cl^−^]_i_ (blue trace, middle) and intracellular acidic transients (red trace, bottom). **(B)** Similar Cl^−^ and H^+^ fluxes were observed when GABA_A_ receptors were activated via stimulation of monosynaptic GABAergic inputs to the ClopHensorN-expressing neuron. **(C)** An ion substitution experiment was performed to confirm independent [Cl^−^]_i_ and pH_i_ measurements using ClopHensorN. Synchronous network events were induced in hippocampal brain slices via bath application of a Mg^2+^-free ACSF. A ClopHensorN-expressing hippocampal neuron was imaged whilst network events were monitored via a whole-cell patch recording from a nearby neuron (<200 μm between somata; black trace, top). The onset of network events (vertical dashed lines) correlated with the onset of intracellular Cl^−^ accumulation (blue trace, middle) and intracellular acidification (red trace, bottom). **(D)** [Cl^−^]_i_ and pH_i_ measurements from the same neuron in “c,” following the replacement of the Mg^2+^-free ACSF with a Cl^−^-free ACSF. Network events (black trace, top) were again correlated with intracellular acidification. However, rather than Cl^−^ influxes, smaller Cl^−^ effluxes were now associated with the network events.

Genetically-encoded reporters of Cl^−^, which are currently available for use within the nervous system, are unable to discriminate between changes in [Cl^−^]_i_ and pH_i_ (Kuner and Augustine, 2000; Markova et al., 2008; Raimondo et al., 2012b). To verify that ClopHensorN is able to dissociate [Cl^−^]_i_ and pH_i_ we performed a series of experiments in ClopHensorN-expressing CA1 and CA3 hippocampal pyramidal neurons, in which we induced different activity-dependent ion fluxes. First, as GABA_A_ receptors are primarily permeable to Cl^−^ and to a lesser extent HCO^−^_3_, strong GABA_A_ receptor activation is predicted to generate a transient increase in [Cl^−^]_i_ and an acidic pH transient due to HCO^−^_3_ efflux (Kaila et al., 1989). Consistent with this prediction, both agonist activation of GABA_A_ receptors by application of GABA (200 μM, 20 ms) and synaptic activation of GABA_A_ receptors via electrical stimulation of GABAergic afferents, resulted in a transient increase in [Cl^−^]_i_ and a modest acidification (Figures 3A,B). Second, we performed ion substitution experiments in which synchronous network activity was elicited by bathing the hippocampal slices in either a Mg^2+^-free ACSF or a Cl^−^-free ACSF; (see Materials and Methods). As the substitution of extracellular Cl^−^ would remove the driving force for this ion to enter cells, we predicted that the two ACSF solutions would significantly alter the ion dynamics measured via ClopHensorN. Whilst imaging the ClopHensorN-expressing pyramidal neurons, synchronous network activity was monitored via a whole cell patch recording from a nearby pyramidal neuron (<200 μm between somata). Consistent with previous reports, network activity in the Mg^2+^-free ACSF generated intracellular acidic transients in the imaged neuron (Raimondo et al., 2012b) and robust accumulation of intracellular Cl^−^ (Figure 3C), presumably due to Cl^−^ infux via activated GABA_A_ receptors (Ilie et al., 2012). In contrast, when the same neuron was imaged in the Cl^−^-free ACSF, synchronous network activity continued to generate acidic transients but the activity-dependent Cl^−^ influxes were now replaced by smaller Cl^−^ effluxes (Figure 3D). This is consistent with the predicted change in transmembrane driving force for Cl^−^. Taken together, these sets of experiments confirmed the ability of ClopHensorN to dissociate changes in Cl^−^ and H^+^ in the nervous system.

### ClopHensorN dissociates activity-dependent Cl^−^ and pH dynamics in hippocampal neurons

**Figure 4:**
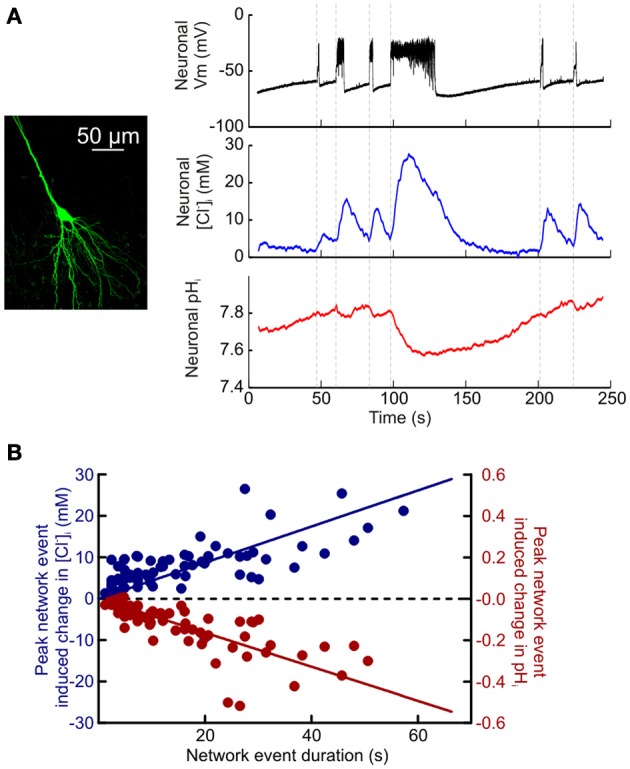
**Quantifying activity-dependent intracellular Cl^−^ and pH dynamics in hippocampal neurons. (A)** Simultaneous measurement of activity-dependent changes in [Cl^−^]_i_ and pH_i_ in a CA3 hippocampal pyramidal neuron expressing ClopHensorN (left). A current clamp recording from a neighboring pyramidal neuron (black trace, top; cell somata <200 μm apart) provided a readout of synchronous network events elicited via bath application of a Mg^2+^-free ACSF. Intracellular Cl^−^ increases (blue trace, middle) and acidic pH shifts (red trace, bottom) were closely associated with periods of heightened network activity (onset indicated by vertical dashed lines). **(B)** Population data depicting the peak recorded shift in [Cl^−^]_i_ (blue) and pH_i_ (red), compared to the duration of the network event (data from 16 neurons). The peak change in [Cl^−^]_i_ was positively correlated with the duration of the network event (*r* = 0.5792, *P* < 0.0001). Whereas the peak change in pH_i_ was negatively correlated with the duration of the network event (*r* = −0.7487, *P* < 0.0001, Pearson correlation).

Having established the potential of ClopHensorN to dissociate simultaneous ion dynamics in the nervous system, we conducted a quantitative study of the magnitude and kinetics of activity-dependent changes in [Cl^−^]_i_ and pH_i_. Synchronous network activity was induced in hippocampal slices by removing Mg^2+^ from the slice perfusate. ClopHensorN-expressing CA1 and CA3 pyramidal neurons were imaged concurrently with whole cell patch-clamp recordings from neighboring cells to provide a simultaneous readout of network activity. Network events resulted in a highly significant positive shift in [Cl^−^]_i_ (*P* < 0.0001, *t*-test, *n* = 75 network events from 16 neurons) and an acidic shift in pH_i_ (*P* < 0.0001, *t*-test, Figures 4A,B). Even short periods of network activity caused detectable shifts in intracellular Cl^−^ and pH (Figure 4A). For instance, network events lasting 1–3 s caused a peak increase in [Cl^−^]_i_ of 2.2 ± 0.7 mM and a 0.017 ± 0.003 pH unit decrease in pH_i_ (*P* = 0.008 and *P* < 0.0001, *t*-test). Furthermore, the magnitude of the peak increase in [Cl^−^]_i_ and decrease in pH_i_ was tightly correlated with the length of the recorded network event ([Cl^−^]_i_: *r* = 0.5792, *P* < 0.0001, pH_i_: *r* = −0.7487, *P* < 0.0001, Pearson Correlation, Figure 4B). The slope of the linear fit revealed a 0.43 mM increase in peak [Cl^−^]_i_ per second of network activity. Conversely, the slope of the linear fit for peak change in pH_i_ revealed a decrease of 0.008 pH units per second of network activity (Figure 4B).

**Figure 5:**
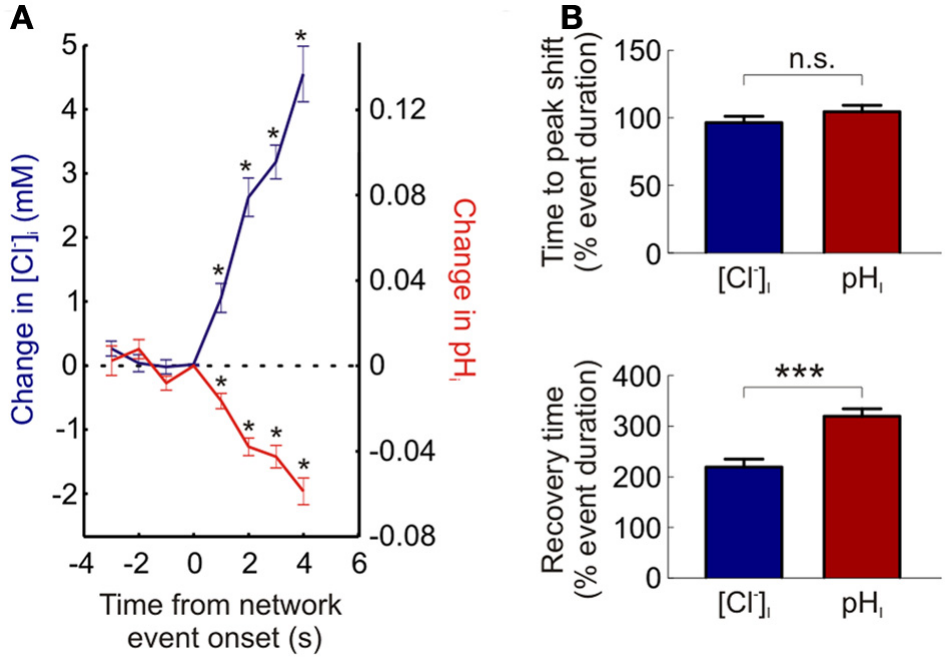
**The kinetics of activity-dependent changes in intracellular Cl^−^ and H^+^. (A)** ClopHensorN allows rapid detection of Cl^−^ and H^+^ changes. The change in [Cl^−^]_i_ (blue, left axis) and pH_i_ (red, right axis) are plotted as a function of the onset of network events (duration >4 s). Significant changes in [Cl^−^]_i_ and pH_i_ (asterisks) were detected within 1.5 s of the onset of elevated network activity (the maximum sample rate; see Materials and Methods). **(B)** Comparing the kinetics of activity-dependent [Cl^−^]_i_ (blue) and pH_i_ (red) shifts in neurons. Whilst there was no significant difference between the time to peak shift in [Cl^−^]_i_ and pH_i_ (top, n.s. *P* = 0.089, *t*-test), activity-dependent [Cl^−^]_i_ changes returned to baseline significantly faster than corresponding pH_i_ transients (bottom, ^***^P < 0.0001, *t*-test). This indicates that separate mechanisms are likely to contribute to the regulation of these two ion species. Error bars indicate SEM.

Utilizing ClopHensorN we were able to detect statistically significant increases in neuronal [Cl^−^]_i_ within 1.5 s of the onset of network activity (the maximum sample rate; see Materials and Methods; *P* = 0.0007, *n* = 37 events, *t*-test, Figure 5A). Likewise intracellular acidification could also be detected within 1.5 s of the onset of elevated network activity (*P* = 0.0007 for neurons, *t*-test, Figure 5A). This demonstrates the rapidity with which intracellular Cl^−^ and pH changes could be monitored using ClopHensorN under our experimental conditions. Lastly, simultaneous, dynamic readout of [Cl^−^]_i_ and pH_i_ allowed us to independently compare the kinetics of activity-dependent changes to neuronal Cl^−^ and pH levels. Whilst the time to peak shift of intracellular ion concentration was not different between the two ions (96.5 ± 4.7 and 104.6 ± 4.7% of network event duration, *P* = 0.089, *t*-test, Figure 5B), [Cl^−^]_i_ recovered to baseline levels significantly faster than pH_i_ (219.6 ± 15.4 vs. 319.8 ± 14.7% of network event duration, *P* < 0.0001, *t*-test, Figure 5B). These experiments demonstrate the utility of using ClopHensorN for quantifying the size and temporal properties of Cl^−^ and H^+^ ion dynamics in neurons.

## Discussion

Here we present ClopHensorN—a genetically-encoded ratiometric Cl^−^ and pH sensor that is optimized for use in the nervous system. ClopHensorN is shown to provide dynamic, simultaneous quantification of intracellular Cl^−^ and H^+^ concentrations under a variety of conditions. Furthermore, our measurements with ClopHensorN have identified previously unrecognized differences in the temporal dynamics of Cl^−^ and pH concentrations, which become evident during periods of network activity. These features distinguish ClopHensorN from currently available reporters and highlight that ClopHensorN represents an important resource for monitoring ion dynamics in the nervous system. This reporter offers new opportunities to understand the regulation and roles played by Cl^−^ and H^+^ in processes such as synaptic transmission, cell morphology changes and metabolism.

ClopHensorN displays the cellular expression profile desired for a ratiometric reporter of intracellular ion concentration and exhibits signal to noise characteristics that are comparable to the best currently available reporters of either Cl^−^ or pH (Raimondo et al., 2012b). ClopHensorN allowed us to detect significant intracellular Cl^−^ and pH changes within our minimum sampling interval (1.5 s). In addition, the reporter displays good photostability with measurements exhibiting negligible drift due to photobleaching, which has been a limitation for other reporters (Bregestovski et al., 2009). These advantageous properties make ClopHensorN suitable for the measurement of both baseline and dynamic changes in Cl^−^ and pH. As with other fluorescent proteins, there is the opportunity to use genetic techniques to target ClopHensorN to specific cell types or subcellular compartments, and to combine the reporter with other molecular tools.

Cl^−^ and H^+^ ions are often co-regulated (Russell and Boron, 1976; Kaila et al., 1989) and neuronal processes can generate fluxes of both ions (Chesler, 2003; Farrant and Kaila, 2007). Therefore, a fundamental advantage of ClopHensorN is that it generates measurements of both ion species and makes it possible to dissociate concentration changes that are occurring simultaneously. This feature of ClopHensorN was confirmed through ion substitution experiments and by detecting the intracellular Cl^−^ and H^+^ accumulation that accompanies intense GABA_A_ receptor activation (Kaila et al., 1989). We then leveraged this capability to make the first simultaneous recordings of activity-dependent intracellular Cl^−^ and pH changes in the hippocampus. ClopHensorN revealed that network activity results in both intracellular Cl^−^ and H^+^ accumulation in neurons, but also revealed differences in the kinetics of these ionic shifts, with pH requiring longer to recover than Cl^−^ changes. This is consistent with the idea that distinct cellular mechanisms contribute to the ongoing regulation of these ions and further illustrates the potential for the reporter to dissociate ion concentrations.

A further advantage of ClopHensorN over other genetic reporters is that it affords measurements of Cl^−^ that are not confounded by pH. Currently, the two most popular genetically-encoded Cl^−^ sensors are Clomeleon and the Clomeleon variant Cl-sensor (Kuner and Augustine, 2000; Markova et al., 2008). Cl^−^ estimates using both of these sensors are affected by pH, which means that concomitant H^+^ fluxes will affect measurements. Using ClopHensorN we provide the first corrected measurements of [Cl^−^]_i_ within hippocampal neurons. Synchronous network activity was observed to cause rapid intracellular Cl^−^ accumulation in neurons. The intraneuronal Cl^−^ accumulation that accompanies synchronous network activity will depolarize the reversal potential for GABA_A_ receptors (Raimondo et al., 2012a). And it has been suggested that this activity-dependent shift in inhibition (“ionic plasticity”) (Rivera et al., 2005; Raimondo et al., 2012c) may regulate NMDA-dependent mechanisms of synaptic plasticity (Staley et al., 1995), improve the coherence of gamma oscillations (Vida et al., 2006) or result in the conversion of physiological activity into pathological epileptiform states (Wright et al., 2011; Lillis et al., 2012).

A disadvantage of ClopHensorN is that it is a reporter by excitation, which means that laser excitation intensity should be measured and correction algorithms used to account for independent fluctuations in the power of confocal laser lines. Lastly, the fact that pH affects the affinity of ClopHensorN for Cl^−^ means that relatively complex procedures (see Materials and Methods) are required to correct Cl^−^ ratios for concurrent pH fluctuations, with the potential for measurement artifacts to occur if performed incorrectly.

Nonetheless, ClopHensorN is the only available genetically-encoded sensor that is able to measure both Cl^−^ and pH within the nervous system—the location for some of the most dynamic changes in intracellular ion concentrations. We foresee future work in which ClopHensorN is used to dissect Cl^−^ and H^+^ fluxes in specific cellular and subcellular compartments, and in the context of different processes in the nervous system.

### Conflict of interest statement

The authors declare that the research was conducted in the absence of any commercial or financial relationships that could be construed as a potential conflict of interest.
